# Universal scaling laws in metro area election results

**DOI:** 10.1371/journal.pone.0192913

**Published:** 2018-02-22

**Authors:** Eszter Bokányi, Zoltán Szállási, Gábor Vattay

**Affiliations:** 1 Department of Physics of Complex Systems, Eötvös Loránd University, Budapest, Hungary; 2 Children’s Hospital, Harvard Medical School, Boston, Massachusetts, United States of America; New England Complex Systems Institute & University of Massachusetts, UNITED STATES

## Abstract

We explain the anomaly of election results between large cities and rural areas in terms of urban scaling in the 1948–2016 US elections and in the 2016 EU referendum of the UK. The scaling curves are all universal and depend on a single parameter only, and one of the parties always shows superlinear scaling and drives the process, while the sublinear exponent of the other party is merely the consequence of probability conservation. Based on the recently developed model of urban scaling, we give a microscopic model of voter behavior in which we replace diversity characterizing humans in creative aspects with social diversity and tolerance. The model can also predict new political developments such as the fragmentation of the left and the immigration paradox.

## Introduction

Formation of cities is the result of socio-economic advantages of concentrating human populations in space outpacing associated costs. Urban agglomeration effects are systematic changes in socio-economic performance, innovation, trade and infrastructure characteristics of all cities as functions of their size. A variety of disciplines including economics [1–3], geography [4, 5], engineering [6] and complex systems [7–9] explain the existence of agglomeration or scaling effects and relate macroscopic properties of a city to its scale (population size). Such relations are known across the sciences as scaling relations [10], and the systematic study of such relationships in cities is known as urban scaling [11–14]. Using the population *N* as the measure of city size, power law scaling takes the form

$$\begin{array}{c} Y=Y_{0}\cdot N^{\beta }, \end{array}$$ (1)

where *Y* can denote material resources such as energy or infrastructure or measures of social activity such as wealth, patents and pollution; *Y*_0_ is a normalization constant. The exponent *β* reflects general dynamic rules at play across the urban system. Similar scale-free, fractal-like behavior has been observed in many human social networks [15] including cities [16]. Therefore, it is natural and compelling that the essential features of a quantitative, predictive theory of cities originate in the dynamics and structure of social [17, 18] and infrastructural networks [19], and that these underlie the observed scaling relations and the values of the exponents [20–22]. In the case of innovation [23], scaling has been related to the long-distance ties that are prevalent in a higher proportion when a larger population provides the potential for productive social interactions.

Most urban socioeconomic indicators have superlinear *β* > 1 exponents and as a result, larger cities are disproportionally the centers of innovation and wealth. Sublinear scaling *β* < 1 characterizes material quantities displaying economies of scale associated with infrastructure, where the agglomeration into cities pays off in having to provide fewer roads, shorter cables etc. Thus, material costs related to living in larger cities is disproportionally low. Linear scaling *β* ≈ 1 is usually associated with individual human needs such as jobs, housing or water consumption [14].

Gomez-Lievano, Patterson-Lomba and Hausmann in Ref. [24] recently proposed a new model (GLPLH model) of superlinear scaling and demonstrated its validity on 43 urban phenomena related to employment, innovation, crime, education and diseases. The model accounts for the difference in scaling exponents and average prevalence across phenomena as well as for the difference in the variance within phenomena across cities of similar size. The central idea is that a number *M* of necessary complementary factors must simultaneously be present for an urban phenomenon to occur. For example, to get a patent at least the following six factors should be present: have a technological problem, have a solution, present the idea clearly, apply for a patent, include subsequent corrections from examiners, and satisfy all the legal requirements.

The fraction of factors that an individual does not have and is expected to require from the city in order to be counted into a phenomenon is *q* ∈ (0, 1), and it quantifies the complexity of that phenomenon. The fraction of factors that a city provides for an individual is *r* ∈ (0, 1). It represents a measure of urban diversity and tends to accumulate logarithmically *r* = *a* + *b* ⋅ log *N* with the population size, where *a* and *b* has been found to be constant across a wide range of urban phenomena. Alternatively, the fraction of factors *not* present in a city is 1 − *r* = *b* ⋅ log *N*_0_/*N*, where log *N*_0_ = (1 − *a*)/*b*, and *N*_0_ ≈ 1.8 ⋅ 10^14^ is a hypothetical maximal diversity attainable in a city. Given a city with *m* factors present, the probability that an individual requires any number of the *m* factors that the city has, but none of the *M* − *m* factors that the city does not have is *P* = (1 − *q*)^*M* − *m*^ ≈ *e*^*q*(*M* − *m*)^ for *q* ≪ 1, and the average number of occurrence of the phenomena is *Y* = *N*〈*P*〉_*N*_, yielding *Y* ≈ *Ne*^*qM*(1 − *r*(*N*))^ = *Ne*^*qMb* log *N*_0_/*N*^ where we used 〈*e*^−*qm*^〉_*N*_ ≈ *e*^−*Mr*(*N*)^ and averaging goes for cities of population *N*. Introducing the the scaling exponent *β* = 1 + *Mbq*, this scaling curve then takes the universal form

$$\begin{array}{c} Y=N_{0}{\left(\frac{N}{N_{0}}\right)}^{\beta }, \end{array}$$ (2)

where *N* is now the part of population conceivably susceptible to the given urban phenomena.

Scaling laws and universality have been observed in various aspects of the political process and elections [25–31], that can be even used to detect election anomalies [32]. In the last decade complex systems-based approaches via social contagion theory has been developed [33–39] for understanding scaling in election data. Here, we concentrate on the phenomenological aspects of the observed scaling only, and don’t study the detailed mechanism behind the process.

In the recent presidential elections in the US it has been noticed that votes for Democrats were disproportionally high in large cities [40], and in the UK major cities also voted to remain in the EU. While this phenomenon can be understood int he context of social contagion, where larger cities are shown to facilitate opinion spreading due to network effect [39], the exact statistical properties of the dependence of the votes on city size can be better explained through scaling laws. Here we show that election data in the US and the referendum votes in the UK show strong evidence of urban scaling. Moreover, in the US, the scaling curves follow a hidden rule, a single parameter family of scaling curves, for both parties and for all the elections in the investigated period of almost 70 years. Using the concept that tolerance and diversity are strongly coupled in cities [41], we develop a microscopic model of voter behavior which produces the macroscopic level urban scaling, explains the observed single parameter scaling, and describes the distribution of deviations from the macroscopic curve. The new model can even explain unexpected voter behaviors like the immigration paradox in Britain, that is, communities that had the fewest recent immigrants from the EU were the most likely in wanting to leave the EU [42].

## Results and discussion

**Fig 1:**
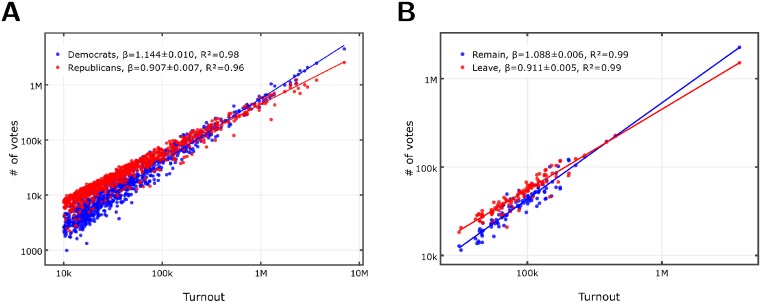
Urban scaling in the presidential elections in the US and in the EU referendum in the UK. Best OLS fit line slopes *β* and regression coefficients *R*^2^ are in the insets (see Section B in S1 File for method details). **A** Doubly logarithmic plot of votes cast for Republicans (red) and Democrats (blue) as the function of the voter turnout for the 912 largest Metropolitan and Micropolitan Statistical Areas of the US in 2016. **B** Doubly logarithmic plot of votes cast for Leave (red) and Remain (blue) as the function of the voter turnout for UK urban electoral districts (see Section B in S1 File for method details).

First, we analyze data for the votes cast for the two main political parties in urban areas in all post-World War II US presidential elections [43] and in the UK EU referendum [44] (see Sections A-B in S1 File for method details). In Fig 1A we show votes for the political options as a function of voter turnout for the 912 largest Metropolitan and Micropolitan Statistical Areas representing about 82% of the total voter population for the 2016 presidential election in the US. Fig 1B shows the votes as a function of voter turnout for the Remain and Leave opinions in the 2016 EU referendum for the urban electoral districts of the UK. The votes for Democrats and Remain in the EU scale superlinearly with exponents *β*_*D*_ ≈ 1.14 and *β*_*rem*_ ≈ 1.09, while votes for Republicans and Leave the EU follow sublinear scaling with *β*_*R*_ ≈ 0.92 and *β*_*lea*_ ≈ 0.91, with high regression coefficients *R*^2^ ≥ 0.9 indicating robust urban scaling. While the elections took place in two different political situations, nevertheless they show very similar exponents.

**Fig 2:**
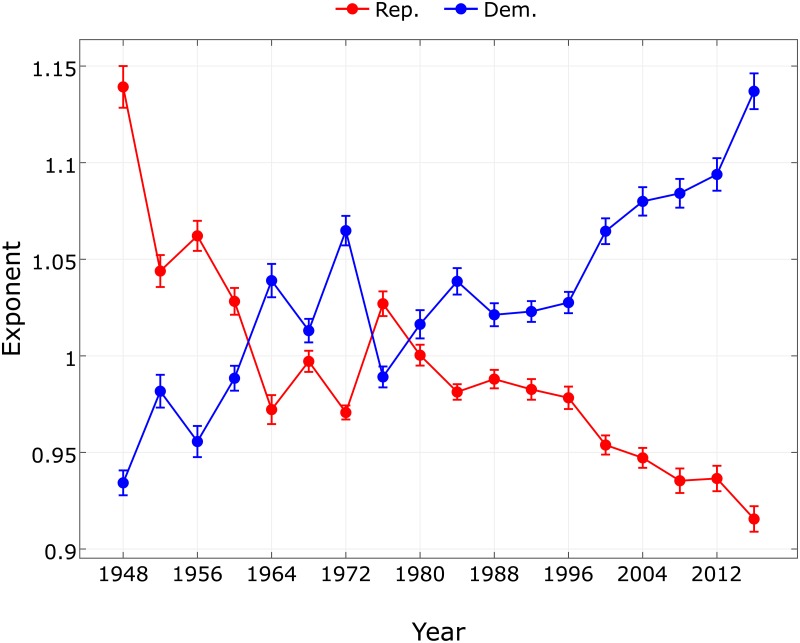
Scaling exponents for the Republicans (red) and Democrats (blue) with error bars for the 18 presidential elections of the US from 1948 to 2016.

**Fig 3:**
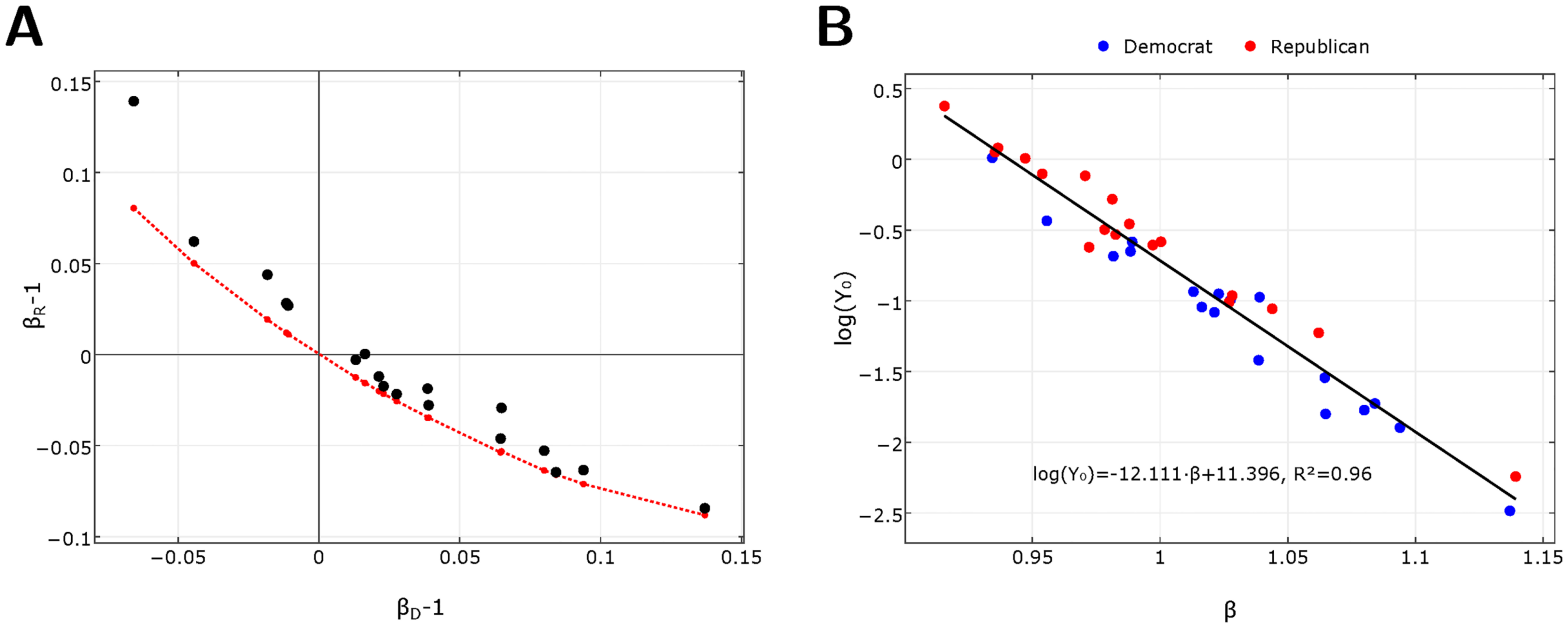
Interrelation of the parameters of urban scaling in US elections. **A** Urban scaling exponents of Republicans as a function of the Democrats for 18 US presidential elections from 1948 to 2016 (dots) and the theoretical curve (red line) derived from probability conservation (5). **B** Intercepts of the scaling relations log *Y*_0_ as a function of the scaling exponent *β* for Republicans (red) and Democrats (blue) for presidential elections in the period 1948–2016. Fitted line (3) with parameters and regression coefficient in the inset.

In Fig 2 we show the historical record of scaling exponents of the Democrats *β*_*D*_ and of the Republicans *β*_*R*_ for the 18 presidential elections in the period 1948–2016. The exponent of the Democrats has an increasing, while the exponent of the Republicans a decreasing historical trend. The Democrat and Republican curves roughly mirror each other in the whole period. The relation of the two exponents becomes apparent when we plot the Republican exponent as a function of the Democrat exponent in Fig 3A.

For each election and for each party we can determine the scaling exponent *β* and the constant *Y*_0_ independently from the fits. In Fig 3B we plot log *Y*_0_ as a function of *β*. We find a very strong (*R*^2^ = 0.96) linear relation

$$\begin{array}{c} \log Y_{0}=-\alpha \beta +\delta , \end{array}$$ (3)

for both parties and for all elections, with *α* = 12.111 and *δ* = 11.396. This indicates that the form of the scaling relation is independent of the party and election and has the universal form

$$\begin{array}{c} Y=e^{\delta -\alpha }N^{*}{\left(\frac{N}{N^{*}}\right)}^{\beta }\approx \frac{1}{2}N^{*}{\left(\frac{N}{N^{*}}\right)}^{\beta }, \end{array}$$ (4)

where *N* is the voter turnout in a city, *β* is the exponent of the party and log N* = *α*. The numerical factor *e*^*δ* − *α*^ is equal to 1/2 within numerical error and the parameter N* ≈ 182,000 is the average turnout of a US city of total population 429,000 in 2016. This is about the size of Fort Wayne IN, the 125th Metropolitan Statistical Area of the US.

The remarkable property of this scaling relation is that in average at turnout *N* = N* the parties share the votes equally (*Y*_*D*_ = *Y*_*R*_ = N*/2) independent of their exponents *β*_*D*_ and *β*_*R*_ or of the year of the election and unaffected by historic changes in population. For cities above turnout N* the party with higher *β* gets the majority of votes, while below this turnout the party with smaller *β* succeeds in average. While in 2016 already 125 metropolitan areas surpassed the population corresponding to this critical turnout, only 45 did so in 1948.

The observed linear relationship (3) and the single parameter form (4) of the scaling curve is predicted by the GLPLH model, therefore, it is reasonable to assume that it can be adapted to the election process. Formally, we recover our scaling curve (4) from this theory by identifying the susceptible population with half of the voter turnout *N*/2 and by setting *N*_0_ = N*/2. There are two discrepancies between our scaling curve (4) and that of the GLPLH model. The GLPLH model is applicable for superlinear *β* > 1 (*Mq* > 0) values only, while in case of elections both superlinear and sublinear exponents arise, and the numerical value of *N*_0_ ≈ 1.8 ⋅ 10^5^ is nine orders of magnitude smaller for elections.

The main difference of elections from other urban phenomena is that the scaling curves influence each other via the competition for votes. This competition is expressed mathematically by the probability conservation *Y*_*D*_/*N* + *Y*_*R*_/*N* = 1 for the sum of the fraction of votes the parties get. Using (4) and averaging for all cities yields

$$\begin{array}{c} \frac{1}{2}\langle {\left(N/N^{*}\right)}^{\beta_{D}-1}\rangle +\frac{1}{2}\langle {\left(N/N^{*}\right)}^{\beta_{R}-1}\rangle =1. \end{array}$$ (5)

This equation guarantees that one of the exponents will be superlinear while the other sublinear (see Section D in S1 File for details). Its numerical solution is shown in Fig 3A. Since we can determine the scaling exponent of one of the parties from the other, a single parameter, the scaling exponent of one of the parties, fully determines the urban scaling curves for both parties. Accordingly, only one of the scaling exponents needs a detailed explanation. A model derived for the results of one of the parties will determine the results of the other party via probability conservation. The strategy of one of the parties will result in a superlinear exponent, which can be explained by an adaptation of the GLPLH model, while the result of the party with the sublinear exponent is just a consequence of the other party’s strategy and the probability conservation law. The explanation of a strategy that results in a superlinear scaling follows later.

Next, we analyze the results in the period 2000–2016, where the Democratic party has a pronounced superlinear scaling, and the Republican party a sublinear scaling. We show that the statistical distribution of the results for Democrats is in accordance with the GLPLH model, while the distribution of the votes for Republicans deviates from it and is merely the consequence of probability conservation. We note here, that while our fits show a superlinear scaling for the Republican party before 1960, the *R*^2^-values of these fits are not as reliable as that of the more recent ones, and therefore, we will continue our analysis of superlinear processes and of the model only for the aforementioned years.

A Scale-Adjusted Metropolitan Indicator [13, 45–48] (SAMI) is the logarithmic deviation of the value *Y*_*i*_ from the average scaling curve for a city with population *N*_*i*_

$$\begin{array}{c} \xi_{i}=\log Y_{i}-\log Y_{0}-\beta \log N_{i}. \end{array}$$ (6)

The articles [13, 46] predict that SAMIs for a given city size range are normally distributed if the investigated measure obeys the urban scaling laws. The GLPLH model states that the SAMI variance can be expressed with the former complexity parameter *q* and the number of complementary factors *M* as $\sigma_{SAMI}^{2}=q^{2}Mb\left(\log N_{0}-\langle \log N\rangle \right),$ where 〈log *N*〉 is the mean of the logarithm of city sizes. It can also be expressed with the scaling exponent

$$\begin{array}{c} \sigma_{SAMI}^{2}=q\left(\beta -1\right)\left(\log N_{0}-\left\langle \log N\right\rangle \right). \end{array}$$ (7)

**Fig 4:**
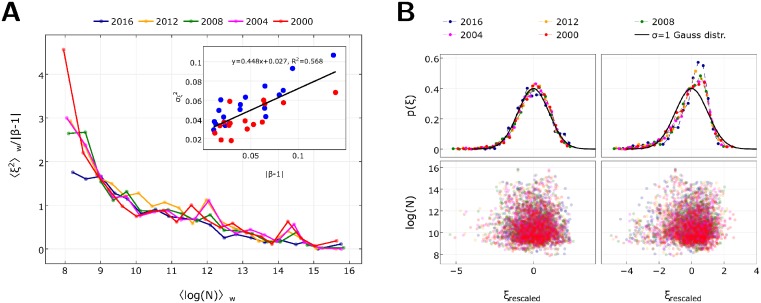
Fluctuations around the average scaling curve. **A**, Variance of the deviation from the average scaling curve as a function of the logarithmic city size, measured in voter turnout. City sizes are binned into 20 windows of uniform sizes on logarithmic scale. In the inset, standard deviation of SAMIs (6) for all metropolitan areas in our study as a function *β* − 1. Best fit line parameters are in the inset. **B**, Standardized deviation of SAMIs for the last five US presidential elections. Lower panel: Scatter plot for the Democrat (left) and Republican (right) standardized deviations (horizontal axis) and logarithmic city size (vertical axis). Upper panel: Distribution of the standardized deviations. For the Democrats (left) it is a standard normal distribution (solid line). For Republicans (right) it is a skewed distribution deviating from the standard normal distribution (solid line). We used the Kolmogorov-Smirnov test to check the normality of the distributions at a significance level of 5% (see Section G in S1 File for details).

In Fig 4 we check the general validity of this formula for both parties and for all elections in the 1948–2016 period. For the variance averaged over all metropolitan areas, though the data is noisy, we cannot reject the notion of proportionality with *β* − 1 (see inset of Fig 4A), indicating also that the complexity parameter *q* is approximately constant over several elections. From the fitted line and from the numerical value 〈log *N*〉 = 10.55 for the 2016 election, we get *q* ≈ 0.28. Then, in the 2000–2016 period for the superlinearly scaling results of the Democrats, we can make a more detailed calculation for ten windows of city sizes. In Fig 4A (main) we can see that curves of $\sigma_{SAMI}^{2}/\left(\beta -1\right)$ for different elections in these windows collapse onto the same curve. While for low city sizes the linear relationship expected after the data collapse does not fit well, for greater city sizes, where statistical errors are lower, the collapse confirms that the complexity parameter is constant. This implies that the change of the scaling exponent *β*_*D*_ for the Democrats in this period comes solely from the change of the number of complementary factors *M*.

Deviations of cities from the average scaling curves can then be standardized in the windows using the window-wise variances. In Fig 4B we show the distribution of these standardized SAMIs for both parties. The distribution of these standardized SAMIs for Democratic party is standard Gaussian, in agreement with the SAMI literature [13, 46]. However, the same procedure results in a skewed distribution for the Republicans. Their rescaled SAMI distribution is not normal and the GLPLH model doesn’t apply. This also confirms that two parties don’t have an equal role in the coupled urban scaling phenomenon.

Now, the question arises, what the necessary complementary factors in the context of elections are that must simultaneously be present in order to vote for Democrats in the 1988–2016 period, where their exponent is superlinear? These factors can be best understood in the terms of issue voting, where voters choose a candidate or an opinion by comparing their own viewpoints in different political issues to that of the voted one [49–51].

We found that the complexity parameter *q* is approximately constant. Thus, from the 4–6 times growth of *β*_*D*_ − 1 in this period we can conclude that the number of issues *M* got multiplicated about 4–6 times. The concrete value of *M* cannot be determined from our data, only the product *bM* that changed from about 0.09 to 0.52 with *b* being constant. If we could find these factors, then Republicans (or the Leave campaign) could be characterized as comprising of those voters, who don’t accept at least one of those *M* issues that are necessary for a voter voting for the superlinearly scaling opinion or party.

Here, we can just conjecture based on the agenda of Democrats, that these are liberal values in general, and the Democrat voter typically accepts all of these *M* values or issues simultaneously. Such values include tolerance and acceptance towards various social groups ranging from women and blacks at the beginning and middle of the 20^th^ century to LGBT communities, immigrants, refugees and various other social minorities recently. In case someone is not able to accept at least one of these, then he or she will probably not vote for the Democrats. That explains why groups of the Republican and the Leave voters look so heterogeneous: they consist of groups that oppose at least one of these liberal values, and that are not held together by a common political agenda otherwise. This also explains the recent steady increase of the Democrat exponent: as more and more *M* values are introduced, it increases the exponent, making bigger cities having disproportionally more Democrats than smaller ones. This result is in line with the findings of [39], who explain the same phenomenon via the increasing impact of social contagion in the more populated areas of the United States.

In this context, we can identify *q* as a probability that a voter—left on its own devices—rejects one of the *M* liberal values, and *r*(*N*) is the probability that a city of size *N* makes a voter tolerant towards those values. Social diversity grows with the city size and voters in cities can face an increasing number of social issues and can develop tolerance towards them. This is in accordance with the immigration paradox in Britain, where voters living near immigrants develop a tolerance, while those who do not are more likely to reject them [42]. Therefore, we expect that just like other types of diversities in cities, tolerance grows like *r*(*N*)∼log *N*/*N*_0_, but the number of maximal social diversity is reached at *N*_0_ ≈ 4 ⋅ 10^5^, which is smaller than the diversity N* ≈ 1.8 ⋅ 10^14^ observed for the more general type of diversity, characterizing humans in creative aspects. Finally, there is one more consequence of this model: as the number of liberal values *M* seems to grow continuously, the potential voters who don’t accept one of them also increases, and becomes detrimental for electoral success. This leads to the fragmentation of the political left, since a larger number of smaller parties accepting only a subset of the *M* values, or even “single-issue” parties can minimize the number of estranged voters and maximize the aggregated votes of all these parties.

## Conclusion

We applied urban scaling theory to the number of votes cast in the Metropolitan and Micropolitan Statistcal Areas in the 1948–2016 presidential elections of the US and the votes cast in the urban areas of the 2016 EU referendum in the UK. We found that out of the two voting options (Democrat/Republican, Remain/Leave), one always follows a superlinear, while the other a sublinear scaling. Using the historical dataset, we showed that instead of four parameters (two for both scaling fits), the single exponent of the superlinearly scaling party is enough to characterize all processes across the elections and the parties. We derived the other exponent from the superlinear exponent by using the conservation of voting probabilities, and showed that the city turnout distribution determines that this other exponent must be sublinear. We then analyzed the fluctuations around the scaling curve distributions and found that the distribution corresponding to the superlinear exponent is lognormal. We concluded that the two parties play different roles in urban scaling. The party with superlinear exponent drives the process, while the scaling of the party with the sublinear exponent is merely the result of probability conservation. In the context of elections we identified the the elements of the GLPLH model and showed that social tolerance and diversity replaces creative diversity in this context. We pointed to new political consequences of the model. We believe that he model and the calculations could further be extended to metropolitan areas in other countries or to electoral systems with multiple choices.

## Materials and methods

See Sections A-G in S1 File for details.

## Supporting information

S1 File Detailed materials and methods. The file contains detailed description of the data sources, fitting procedures (power laws and the Kolmogorov-Smirnov test) and calculations.
(PDF)
