# Supplementary material for: Universal scaling laws in metro area election results
Source: PLoS One. 2018 Feb 22;13(2):e0192913. doi: 10.1371/journal.pone.0192913 (PMC5823399; doi:10.1371/journal.pone.0192913)
Supplement: S1 File — The file contains detailed description of the data sources, fitting procedures (power laws and the Kolmogorov-Smirnov test) and calculations. (PDF) [file pone.0192913.s001.pdf]

A Data sources

We downloaded county-level historical US presidential election datasets from [1]. We calculated the total number of votes for the Democratic and Republican Party and the turnouts for all Metropolitan and Micropolitan Statistical Areas [2] by matching MSA's to the county level data [3].

As for the UK, we downloaded electorate-level number of votes for Remain and Leave from the EU referendum result dataset [4]. We filtered the UK electorates based on whether they have a city in their core [5], because the resolution of the data available about the referendum was not enough to consider using cities as units.

B Data fit

For each year y, we assume that the expected value of the number of voters for a party (D, Democrat or R, Republican) scales with the size of a city in the following way:

$$Y^{(y)}(N) = Y_{0,D/R}^{(y)} \cdot N^{\beta_{D/R}^{(y)}}.$$

Taking the logarithm of both sides, we can fit a line using OLS fit on the $(\log Y, \log N)$ pairs for each election for both parties (we leave the year and party notations for simplicity reasons):

$$\log(Y(N)) = \log(Y_0) + \beta \cdot \log(N),$$

where the β denotes the slope, $\log Y_0$ the the intercept of the fitted line, thus β is the exponent of the party in year y.

C Pivotal point

If we assume that the intercept $\log(Y_0)$ is a function of β that changes slowly with β, and we know that β is always close to 1, then we can approximate $\log Y_0$ around 1 linearly:

$$\log(Y_0(\beta)) \approx \underbrace{\log(Y_0(1))}_{\delta-\alpha} + (\beta - 1) \underbrace{\left.\frac{\partial \log(Y_0(\beta))}{\partial \beta}\right|_{\beta=1}}_{-\alpha} + \cdots = -\alpha \cdot \beta + \delta$$

In the case of $\beta = 1$, it has to be true, that

$$Y_0(1) = e^{\delta-\alpha} = \langle p \rangle = p_0,$$

the city-averaged voter fractions, because that would mean that every city votes as if all voters were dispersed homogeneously:

$$Y_D(N) = p_0 N.$$

Let $\alpha = \log N^*$, then $p_0 = e^\delta / e^\alpha = e^\delta / N^*$.

$$\log(Y_0(\beta)) = -\log N^* \cdot \beta + \log(p_0) + \log N^*$$

By substituting it into the original scaling relation:

$$\log Y(N) = \log(p_0 N^*) - \beta \cdot \log N^* + \beta \cdot \log N$$

thus,

$$Y(N) = p_0 N^* \left(\frac{N}{N^*}\right)^\beta = p_0 N \left(\frac{N}{N^*}\right)^{\beta - 1}$$

This implies that all fitted lines have to go through the $(N^*,\ p_0 N^*)$ point, because at $N = N^*$, Y equals to $p_0 N^*$ regardless of the value of β. Also note, that N^* is universal for both parties and for all elections. Thus, the scaling relations only have only parameter, the scaling exponent β.

D Exponent relationship

In a given year, for every city i it holds that the number of Democrat and Republican voters is approximately equal to the turnout in the city:

$$\frac{Y_D^{(i)}}{N^{(i)}} + \frac{Y_R^{(i)}}{N^{(i)}} = 1$$

Assuming scaling, the expected values of the Democrat and Republican voters can be substituted:

$$Y_D^{(i)} = \frac{1}{2} N^* \left(\frac{N^{(i)}}{N^*}\right)^{\beta_D} = \frac{1}{2} N^{(i)} \left(\frac{N^{(i)}}{N^*}\right)^{\beta_D - 1}$$
$$Y_R^{(i)} = \frac{1}{2} N^* \left(\frac{N^{(i)}}{N^*}\right)^{\beta_R} = \frac{1}{2} N^{(i)} \left(\frac{N^{(i)}}{N^*}\right)^{\beta_R - 1}$$

Thus,

$$\frac{1}{2}\left(\frac{N^{(i)}}{N^*}\right)^{\beta_D-1}+\frac{1}{2}\left(\frac{N^{(i)}}{N^*}\right)^{\beta_R-1}=1$$

$$\left(\frac{N^{(i)}}{N^*}\right)^{\beta_D-1}+\left(\frac{N^{(i)}}{N^*}\right)^{\beta_R-1}=2$$

Because the exponents β_D and β_R are close to 1, the left hand side can be approximated to the second order

$$1+(\beta_D-1)\cdot\log\frac{N^{(i)}}{N^*}+\frac{1}{2}(\beta_D-1)^2\cdot\left(\log\frac{N^{(i)}}{N^*}\right)^2+\cdots+1+(\beta_R-1)\cdot\log\frac{N^{(i)}}{N^*}+\frac{1}{2}(\beta_R-1)^2\cdot\left(\log\frac{N^{(i)}}{N^*}\right)^2+\cdots=2$$

Let us average the equation over all cities in a year:

$$(\beta_D-1)\cdot\left\langle\log\frac{N^{(i)}}{N^*}\right\rangle+\frac{1}{2}(\beta_D-1)^2\cdot\left\langle\left(\log\frac{N^{(i)}}{N^*}\right)^2\right\rangle+(\beta_R-1)\cdot\left\langle\log\frac{N^{(i)}}{N^*}\right\rangle+\frac{1}{2}(\beta_R-1)^2\cdot\left\langle\left(\log\frac{N^{(i)}}{N^*}\right)^2\right\rangle=0$$

In the first order, $\beta_R-1=-(\beta_D-1)$. Because the term $(\beta_R-1)^2$ is small, we only use its first order approximation, thus:

$$(\beta_D-1)\cdot\left\langle\log\frac{N^{(i)}}{N^*}\right\rangle+\frac{1}{2}(\beta_D-1)^2\cdot\left\langle\left(\log\frac{N^{(i)}}{N^*}\right)^2\right\rangle+(\beta_R-1)\cdot\left\langle\log\frac{N^{(i)}}{N^*}\right\rangle+\frac{1}{2}(-(\beta_D-1))^2\cdot\left\langle\left(\log\frac{N^{(i)}}{N^*}\right)^2\right\rangle=0$$

$$(\beta_D-1)\cdot\left\langle\log\frac{N^{(i)}}{N^*}\right\rangle+(\beta_D-1)^2\cdot\left\langle\left(\log\frac{N^{(i)}}{N^*}\right)^2\right\rangle+(\beta_R-1)\cdot\left\langle\log\frac{N^{(i)}}{N^*}\right\rangle=0$$

$$\beta_R-1=-(\beta_D-1)\cdot+(\beta_D-1)^2\cdot\frac{\left\langle\left(\log\frac{N^{(i)}}{N^*}\right)^2\right\rangle}{\left\langle\log\frac{N^{(i)}}{N^*}\right\rangle}$$

E EU referendum UK 2016

Similarly to that of the presidential election dataset in the United States, we fitted the $Y=Y_0\cdot N^\beta$ function on the number of Remain and Leave votes for the EU referendum in the cities of the United Kingdom. Electorate-level data was obtained from the homepage of the Electoral Commission [6]. Since we did not have a city-level resolution, we took electorates that were centered around a city, and used only their turnouts as N, and number of voters as Y.

Because the distribution of city sizes in the UK is very uneven even on the logarithmic scale with London being disproportionally large, we weighted the points by $1/N$ in the OLS fit on the double logarithmic plot.

As in the case of the US Democrats, the Remain votes showed a strong superlinear scaling with $\beta_{\text{Remain}} = 1.08$, while the Leave votes scale sublinearly $\beta_{\text{Leave}} = 0.91$

F Turnout scaling

For the elections where we had both population and turnout data, we fitted the equation (see Section 2)

$$Y^{(y)}(N) = Y_0^{(y)} \cdot N^{\beta_T},$$

where this time Y denotes the turnout of the election in year y, and N denotes the actual population of a city. An actual fit for the 2016 election is shown in Figure 2.

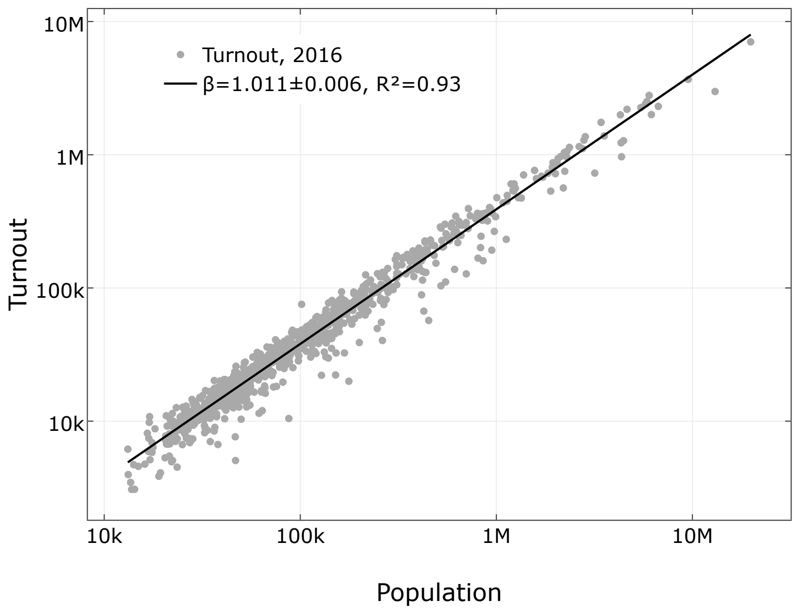

Figure 1: Scaling of turnout with city population in the 2016 US presidential election.

The historical exponent values are plotted in Figure 3.

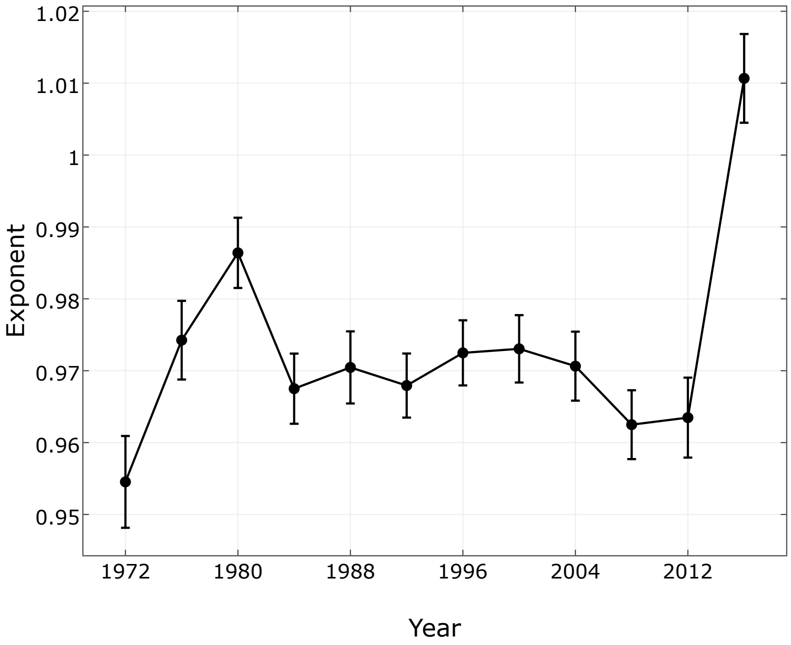

Figure 2: Historical scaling exponents of turnout fits in US presidential elections.

G Kolmogorov-Smirnov test

We tested the rescaled SAMI distributions of the Democrat and Republican fits in the elections 2000-2016 for normailty. The below table shows the p-values of the tests, based on which normality can be rejected at a significance level of 5% for the Republican SAMI distributions, whereas it cannot be rejected for the Democrat SAMI distributions. The distributions are shown in Fig. 4B of the manuscript.

Year	p_{Dem}	p_{Rep}
2000	0.0675	2.23e-03
2004	0.1090	3.14e-04
2008	0.1670	2.30e-04
2012	0.0746	2.05e-07
2016	0.8440	7.04e-13

Table 1: **p-values for the Kolmogorov-Smirnov test on the distribution of the rescaled SAMIs.** p_{Dem} shows the p-values for the different election years for the Democrat rescaled SAMI distributions, while p_{Rep} shows the same for the Republicans.

References

[1] Leip D. Dave Leip U.S. Presidential General County Election Results. Harvard Dataverse; 2016. Available from: http://dx.doi.org/10.7910/DVN/SUCQ52.

[2] Metropolitan and Micropolitan Statistical Areas Main - US Census Bureau 2016; Date accessed: 2016-12-28. https://www.census.gov/population/metro/.

[3] CBSA to FIPS County Crosswalk; Date accessed: 2017-02-09. http://www.nber.org/data/cbsa-fips-county-crosswalk.html.

[4] Electoral Commission; Date accessed: 2016-12-28. http://electoralcommission.org.uk.

[5] United Kingdom: Countries and Major Cities - Population Statistics in Maps and Charts; Date accessed: 2016-12-28. https://www.citypopulation.de/UK-Cities.html.

[6] EU referendum results, The Electoral Commission (UK); Date accessed: 2017-02-09. http://www.electoralcommission.org.uk.
